# Platelet status in cancer cachexia progression in Apc^Min/+^ mice

**DOI:** 10.3389/fimmu.2023.1253587

**Published:** 2023-08-28

**Authors:** Patrice Cunningham, Christian A. Unger, Emma A. Patton, Akyla Aiken, Alea Browne, Ella James, Ahmed K. Aladhami, Marion C. Hope 3rd, Brandon N. VanderVeen, Thomas D. Cardaci, E. Angela Murphy, Reilly T. Enos, Kandy T. Velázquez

**Affiliations:** ^1^ Department of Pathology, Microbiology, and Immunology, School of Medicine, University of South Carolina, Columbia, SC, United States; ^2^ Columbia Department of Veterans Affairs Health Care System, Columbia, SC, United States

**Keywords:** muscle wasting, platelets, physical activity, red blood cells, TGFβ

## Abstract

Cachexia, a complex wasting syndrome, significantly affects the quality of life and treatment options for cancer patients. Studies have reported a strong correlation between high platelet count and decreased survival in cachectic individuals. Therefore, this study aimed to investigate the immunopathogenesis of cancer cachexia using the Apc^Min/+^ mouse model of spontaneous colorectal cancer. The research focused on identifying cellular elements in the blood at different stages of cancer cachexia, assessing inflammatory markers and fibrogenic factors in the skeletal muscle, and studying the behavioral and metabolic phenotype of Apc^Min/+^ mice at the pre-cachectic and severely cachectic stages. Platelet measurements were also obtained from other animal models of cancer cachexia - Lewis Lung Carcinoma and Colon 26 adenocarcinoma. Our study revealed that platelet number is elevated prior to cachexia development in Apc^Min/+^ mice and can become activated during its progression. We also observed increased expression of TGFβ2, TGFβ3, and SMAD3 in the skeletal muscle of pre-cachectic Apc^Min/+^ mice. In severely cachectic mice, we observed an increase in Ly6g, CD206, and IL-10 mRNA. Meanwhile, IL-1β gene expression was elevated in the pre-cachectic stage. Our behavioral and metabolic phenotyping results indicate that pre-cachectic Apc^Min/+^ mice exhibit decreased physical activity. Additionally, we found an increase in anemia at pre-cachectic and severely cachectic stages. These findings highlight the altered platelet status during early and late stages of cachexia and provide a basis for further investigation of platelets in the field of cancer cachexia.

## 1 Introduction

Cachexia is defined as an unintentional body weight loss, which can include loss of muscle with or without loss of fat mass (1, 2). The progression of cancer cachexia is often classified into stages – pre-cachexia, cachexia, and severe/refractory cachexia – based on weight loss, muscle functional capacity, and inflammatory status (1). Despite interventions, reversing or halting the progression of cachexia is extremely challenging. Therefore, cachexia contributes to an increased mortality rate (3, 4). Moreover, a study conducted in cancer patients found weight loss was a predictor of mortality (5). In addition, a multi-institutional observational study in cancer patients found that factors such as body weight loss, decreased handgrip strength, anorexia, and fatigue are associated with the deterioration of quality of life resulting from the progression of cancer cachexia (6). Currently, there are no approved therapies for cancer cachexia, and drugs targeting its inflammatory component have not respond as expected (7). Exploring new processes, such as the immunopathogenesis of cachexia, may provide insights into its initiation and facilitate the development of drugs to counter muscle wasting.

There is evidence to suggest that platelets, small anucleate cells which participate in hemostasis as well as inflammatory and immunological processes, may be involved in the development of cachexia in humans (8–16). Notably, a strong association has been observed between high platelet counts and decreased survival in cachectic patients (12, 17, 18). Research has shown that platelets release a variety of molecules that can contribute to the inflammation and tissue wasting seen in cachexia (19). These molecules include cytokines, chemokines, and growth factors that can activate pathways involved in muscle breakdown and inhibit pathways involved in muscle growth (20–22). In addition, platelets can interact with immune cells such as macrophages and neutrophils, which are also involved in the development of cachexia (23–25). Platelets can stimulate the release of pro-inflammatory molecules from these cells, exacerbating the inflammatory response seen in cachexia. Furthermore, platelets can also promote the release of various growth factors linked to fibrosis (26). These facts, together with epidemiological data, provide evidence of a strong association between cancer mortality and an increased platelet count (17, 18, 27).

Elevated platelet levels are consistently observed in individuals with a poor prognosis in cancer (28–31). A large case-control study reported a significant association between a very high platelet count and an increased risk of developing colon, lung, and stomach cancers within three years (32). These cancers are the malignancies with the highest cachexia incidence (4, 33). Experimental models of colorectal cancer have shown that platelet sequestration in the spleen occurs prior to the elevation of the inflammatory cytokine interleukin-6 (IL-6) (34). It has also been demonstrated that IL-6 can stimulate thrombopoietin production, which leads to platelet formation (35, 36). Other studies suggest the clinical relevance of modifying platelet responses in inflammatory conditions, as antiplatelet medications have been shown to reduce mortality from infections and sepsis (37, 38). Therefore, it is possible that platelets might be involved in the initiation as well the severity of cancer cachexia.

The purpose of this study was to investigate the immunopathogenesis of cancer cachexia using the preclinical model of spontaneous colorectal cancer, Apc^Min/+^ mice. First, we sought to identify cellular elements of the blood at different stages of cancer cachexia. A complete blood count (CBC) is a commonly conducted test in humans due to its usefulness in assessing various health conditions (39). It is particularly valuable in evaluating cell components of the blood, including white blood cells, red blood cells, and platelets. Subsequently, we assessed inflammatory markers and fibrogenic factors in the skeletal muscle. Furthermore, we investigated the behavioral and metabolic phenotype of Apc^Min/+^ mice at the pre-cachectic stage. Lastly, platelet measurements were obtained from two other mouse models of cancer cachexia, Lewis Lung Carcinoma and Colon 26 adenocarcinoma, to determine if platelet number increases and/or activation occurs in other animal models of cancer cachexia.

## 2 Materials and methods

### 2.1 Animals

C57BL/6J (strain #000664) and C57BL/6J-Apc^Min^/J (Apc^Min/+^, strain #002020) were originally purchased from The Jackson Laboratory (Bar Harbor, ME, USA). C57BL/6J female mice were bred with C57BL/6J-Apc^Min^/J male mice to obtain age- (17-44 weeks of age) and sex-matched littermate controls. C57BL/6J (male and female) and Apc^Min/+^ (male and female) mice were weaned and housed together in ventilated cages to minimize environmental and microbiome-related differences. Lewis Lung Carcinoma or LLC (ATCC, Manassas, VA, USA; CRL-1642) cells were implanted (1X 10^6^, 3^rd^ passage) in the right flank area of C57BL/6J male mice (16-20 weeks of age) age purchased from Jackson Laboratory. Colon 26 adenocarcinoma or C26 cells (donated from Dr. Andrea Bonetto) were implanted (1X 10^6^, 3^rd^ passage) at 15 weeks of age in the subscapular region of CD2F1 male mice (Charles River Laboratory, Raleigh NC, USA). CD2F1 mice were euthanized two weeks after C26 cell implantation. The breeding protocol and experiments conducted in this study were approved by the Institutional Animal Care and Use Committee (IACUC) of the University of South Carolina. Mice were fed purified AIN-76A diet *ad libitum* (Bio-Serv, Frenchtown, NJ, USA) and room lighting followed a 12-hour light and 12-hour dark cycle with a temperature average of 22°C and humidity of 50%. Weekly body weight measurements were recorded to assess body weight loss from their peak body weight. Apc^Min/+^ mice with less than 5% body weight loss was assigned to the pre-cachectic group, while those with body weight loss exceeding 10% were allocated to the severely cachectic group. Both groups, along with C57BL6/J littermate control mice, were euthanized with an overdose of isoflurane once the desired body weight loss was reached in Apc^Min/+^ mice. Blood and tissue samples were collected for further analysis. LLC-tumor bearing mice were euthanized twenty-five days post-implantation and stratified based on body weight loss after tumor excision. Meanwhile, C26-tumor bearing mice were euthanized two weeks after tumor implantation and exhibited >10% body weight loss after tumor removal.

### 2.2 Behavioral and metabolic phenotyping

Behavioral and metabolic phenotyping of C57BL/6J (n=9) and Apc^Min/+^ pre-cachectic (n=7) mice were performed between twelve and fifteen weeks of age for one week using the Promethion System (Sable System International, Las Vegas, NV, USA) as previously described (40). Severely cachectic Apc^Min/+^ mice were not included in this assessment as survival was not an end point in this experiment. Food and water consumption, body mass, locomotor activity, energy expenditure, and sleeping patterns during the light and dark cycle were collected and analyzed. Metabolic parameters were analyzed using the lean body mass (Dual-Energy X-ray Absorptiometry) as a covariate.

### 2.3 Functional test

Forelimb strength and neuromuscular function of C57BL/6J (n=9) and Apc^Min/+^ pre-cachectic (n=7) mice were determined using grip strength (Bioseb, Pinellas Park, FL, USA) and rotarod (Panlab, Harvard Apparatus, Holliston, MA) assessments. For grip strength, mice were placed in a grid and their tail was gently pulled backward until they lost their grip. The average peak force from five trials was recorded and graphed. In the neuromuscular function test, mice were habituated to the rotarod, which is an elevated rotating rod. On the test day, mice were placed on the rod and subjected to a ramping protocol (0-25 rpm, 0.02 x g) over a period of 120 sec or until they fell from the rod. This protocol was repeated three times with a 2-minute waiting interval. The best performance time was recorded and graphed.

### 2.4 Complete blood count

Blood collected during euthanasia was analyzed using the VetScan Hm5 (Abaxis, Union City, CA, USA) for hematology assessment. The complete blood count and 3-part differential were utilized to measure various parameters, including white blood cells (WBC), lymphocytes (LYM), monocytes (MON), neutrophils (NEU), red blood cells (RBC), Hemoglobin (HGB), hematocrit (HCT), mean corpuscular volume (MCV), mean corpuscular hemoglobin (MCH), red cell distribution width (RDW), platelets (PLT), mean platelet volume (MPV), and platelet distribution width (PDWs).

### 2.5 Gastrointestinal polyp quantification

Small and large intestines were dissected and rinsed in PBS. Polyp numbers were counted under a dissection microscope after euthanasia.

### 2.6 Quantitative real time PCR

The gastrocnemius muscle of Apc^Min/+^mice and C57BL6/J littermate controls was homogenized in Trizol for RNA extraction. The isolated RNA was quantified using a nanodrop, and the iScript cDNA synthesis kit was used to transcribe and analyze the expression of specific genes, including: Ly6g, F4/80, CD206/mrc1, IL-10, IL-1β, IL-4, IL-6, TNFα, TGFβ1, TGFβ2, TGFβ3, SMAD2, SMAD3, and fibrinogen. Reference genes (Nono, Gusb, Hmbs, RplpO, 18S, H2afv) were also run, and stable genes (Nono, Hmbs, RplpO, H2afv) were selected as the normalization factors for each analysis using Qbase + software (Biogazelle, Ghent, Belgium).

### 2.7 Statistical analysis

The data were analyzed using Prism 8 (GraphPad Software, La Jolla, CA). One-way ANOVA or mixed-effects model tests were performed, followed by Tukey’s multiple comparison test or Sidak’s multiple comparisons test, respectively. Unpaired two-tailed t-tests were used to compare the number of polyps between pre-cachectic and severely cachectic Apc^Min/+^ mice, as well as functional tests (grip strength and rotarod test) between C57BL/6J and Apc^Min/+^ pre-cachectic mice, and platelet count between non-cancer and tumor-bearing mice. If Bartlett’s test for equal variance failed, the data were log-transformed and reanalyzed. Metabolic parameters were analyzed using an ANCOVA (MMPC National Mouse Metabolic Phenotyping Center) (41) with lean body mass as a covariate. The data are presented as means ± standard error, and a significance level of p<0.05 was used.

## 3 Results

### 3.1 Skeletal muscle and fat mass changes with cancer cachexia progression in Apc^Min/+^ male and female mice

**Figure 1:**
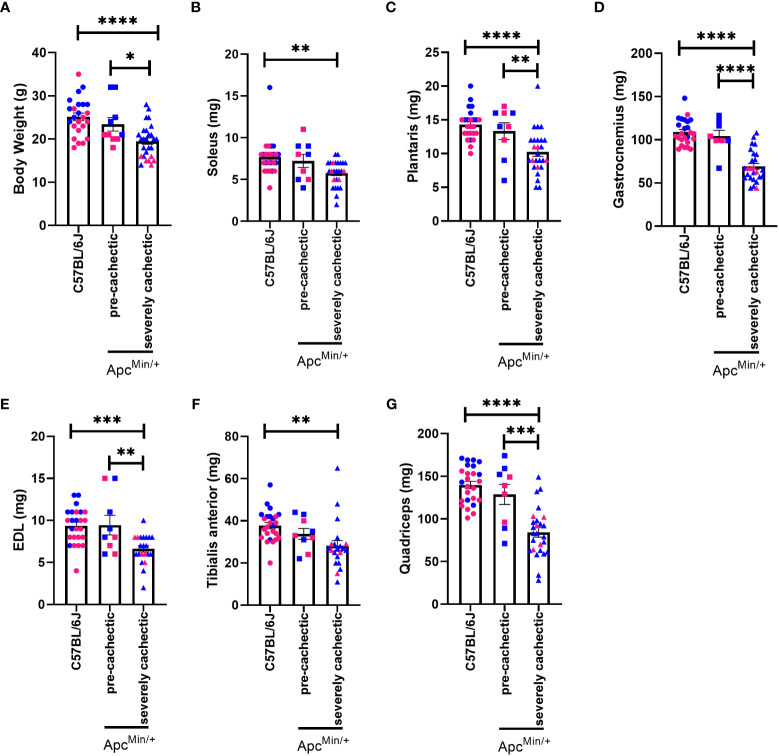
Body weight and hindlimb muscle mass in male and female Apc^Min/+^ mice during cancer cachexia progression. **(A)** Body weight **(B)** soleus, **(C)** plantaris, **(D)** gastrocnemius, **(E)** EDL, **(F)** TA, and **(G)** quadricep muscle mass of at the end point of the experiment in non-cancer control C57BL/6J (n=24), pre-cachectic Apc^Min/+^ (n=10, body weight loss <5%), and severely cachectic (n=27, body weight loss >10%) mice. Pink represents females, and blue represents male mice. One-way ANOVA followed by a Tukey’s multiple comparisons test. *p<0.05, **p<0.01, ***p<0.001, and ****p<0.0001.

To confirm the progression of cancer cachexia, we measured body weight, polyp number, as well as mass of hindlimb muscles, spleen, liver, and fat. As expected, severely cachectic Apc^Min/+^ mice (with an average body weight loss of twenty-four percent) weighed significantly less than Apc^Min/+^ pre-cachectic (with an average body weight loss of four percent) and C57BL/6J mice at euthanasia (**Figure 1A**, p=0.0425 and p<0.0001). The mass of the soleus, plantaris, gastrocnemius, extensor digitorum longus (EDL), tibialis anterior, and quadriceps muscles was also reduced in severely cachectic Apc^Min/+^ mice compared to C57BL/6J mice (**Figures 1B–G**, p=0.0022, p<0.0001, p<0.0001, p=0.0004, p=0.0028, and p<0.0001 respectively). Moreover, significant differences in the mass of the plantaris, gastrocnemius, EDL, and quadriceps were observed between pre-cachectic Apc^Min/+^ and severely cachectic Apc^Min/+^ mice (p=0.0228, p<0.0001, p=0.0079, and p=0.0002 respectively). Splenomegaly was observed in both pre-cachectic Apc^Min/+^ and severely cachectic Apc^Min/+^ mice when compared to C57BL/6J mice (**Table 1**, p=0.0029 and p<0.0001). No significant difference in liver weight was observed between the groups. There was no difference in fat pad mass between C57BL/6J mice and pre-cachectic Apc^Min/+^ mice. However, a significant reduction in gonadal and retroperitoneal fat masses were observed between severely cachectic Apc^Min/+^ mice and C57BL/6J mice (p=0.0010 and p=0.0032). No significant differences were observed in fat pad mass and polyp number between pre-cachectic Apc^Min/+^ and severely cachectic Apc^Min/+^ mice. Similarly, previous studies have shown no changes in polyp size relative to cachectic stage (42–46).

**Table 1:** Organ weight, fat pad mass, and polyp number in pre-cachectic and cachectic mice.

Group	Spleen (mg)	Liver (gr)	Gonadal fat (mg)	Retroperitoneal fat (mg)	Polyp number
C57BL/6J	81.5 ± 6.0	0.990 ± 0.05	589 ± 108	232 ± 45	N.T.
Apc^Min/+^ pre-cachectic	258.9 ± 40.1*	1.120 ± 0.10	319 ± 27	99 ± 13	27 ± 9
Apc^Min/+^ severely cachectic	358.5 ± 37.1*	1.145 ± 0.07	11 ± 8*	18 ± 8*	28 ± 3

*p<0.05.

### 3.2 Hematological profile in cancer cachexia progression in Apc^Min/+^ male and female mice

**Figure 2:**
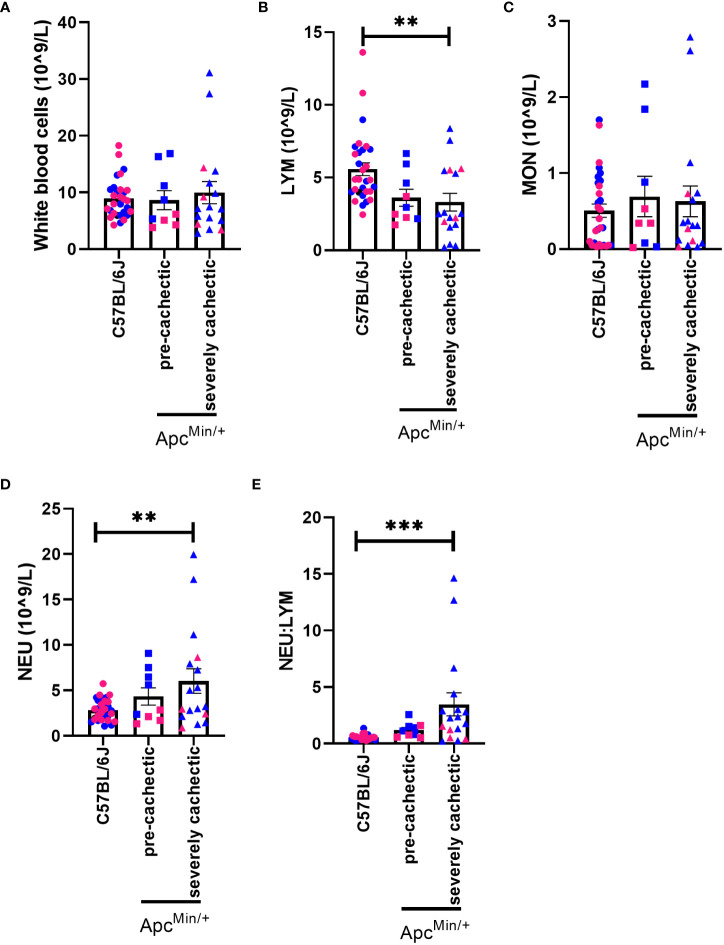
White blood cells profile in male and female Apc^Min/+^ mice during cancer cachexia progression. **(A)** White blood cell, **(B)** lymphocyte, **(C)** monocyte, **(D)** neutrophil, and **(E)** neutrophil:lymphocyte in the blood of non-cancer control C57BL/6J (n=24), pre-cachectic Apc^Min/+^(n=9), and severely cachectic Apc^Min/+^ mice (n=17). Pink represents females, and blue represents male mice. One-way AVONA followed by a Tukey’s multiple comparisons test. **p<0.01 and ***p<0.001.

To characterize the hematological profile during cancer cachexia progression, we measured the number of white blood cells, red blood cells, and platelets in the blood of pre-cachectic Apc^Min/+^, severely cachectic Apc^Min/+^, and C57BL/6J mice. The total number of white blood cells did not differ between groups (**Figure 2A**). However, severely cachectic Apc^Min/+^ mice showed a significant reduction in lymphocyte number compared to C57BL/6J mice (p=0.0071), but not compared to pre-cachectic Apc^Min/+^ mice (**Figure 2B**). There was no difference in monocytes between groups (**Figure 2C**). A significant increase was observed in the number of neutrophils in severely cachectic Apc^Min/+^ mice compared to C57BL/6J mice (p=0.0082), but not compared to pre-cachectic Apc^Min/+^ mice (**Figure 2D**). When the neutrophil:lymphocyte was calculated, a significant increase was observed in severely cachectic Apc^Min/+^ mice compared to C57BL/6J mice (**Figure 2E**, p=0.0003). There was a trend towards an increase neutrophil:lymphocyte in pre-cachectic Apc^Min/+^ compared to C57BL/6J mice (p=0.0550).

**Figure 3:**
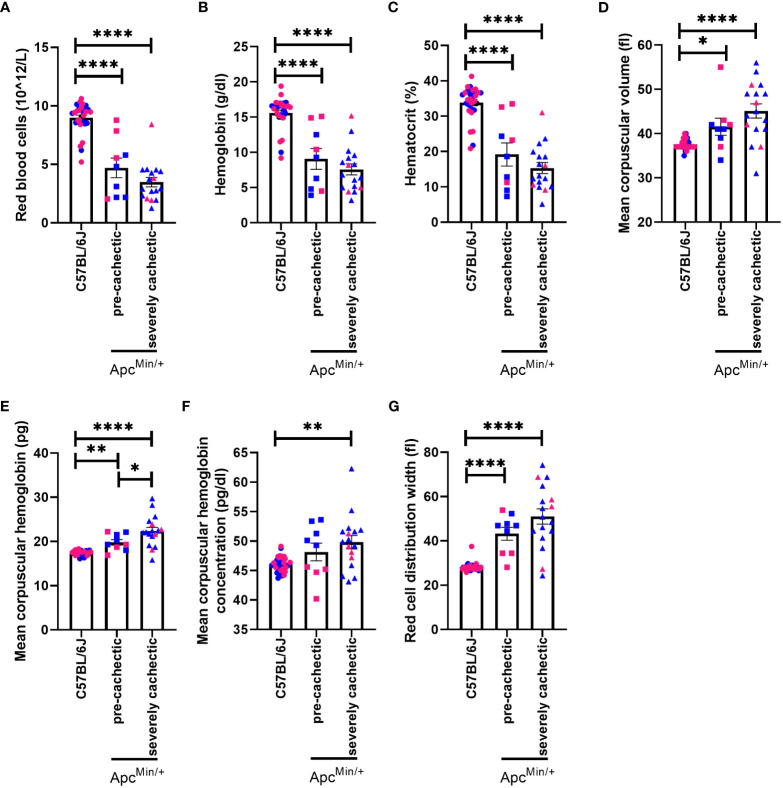
Hematological profile in male and female Apc^Min/+^ mice during cancer cachexia progression. **(A)** Red blood cell **(B)** hemoglobin **(C)** hematocrit, **(D)** mean corpuscular volume, **(E)** mean corpuscular hemoglobin, **(F)** mean corpuscular hemoglobin concentration **(G)** red blood cell distribution width in the blood of non-cancer control C57BL/6J (n=24), pre-cachectic Apc^Min/+^ (n=9), and severely cachectic Apc^Min/+^ mice (n=17). Pink represents females, and blue represents male mice. One-way AVONA followed by a Tukey’s multiple comparisons test. *p<0.05, **p<0.01, and ****p<0.0001.

Anemia is one of the hallmarks of cancer cachexia (47). Therefore, we proceeded to identify if anemia precedes muscle mass loss. A reduction in the number of red blood cells, hemoglobin, and hematocrit was observed in both pre-cachectic Apc^Min/+^ and severely cachectic Apc^Min/+^ mice compared to C57BL/6J mice (**Figures 3A–C**, p<0.0001, p<0.0001, and p<0.0001). Additionally, an increase in red blood cell indices was observed between Apc^Min/+^ and C57BL/6J mice (**Figures 3D–G**). Mean corpuscular volume, or the size of the red blood cell, was significantly increased in pre-cachectic Apc^Min/+^ and severely cachectic Apc^Min/+^ mice compared to C57BL/6J mice (**Figure 3D**, p=0.0473 and p<0.0001). Mean corpuscular hemoglobin, or the amount of hemoglobin per red blood cell, was significantly elevated in pre-cachectic Apc^Min/+^ and severely cachectic Apc^Min/+^ mice compared to C57BL/6J mice (**Figure 3E**, p=0.0085 and P<0.0001). Additionally, a difference in mean corpuscular hemoglobin was observed between pre-cachectic and severely cachectic Apc^Min/+^ mice (p=0.0127). An increase in the average concentration of hemoglobin per red blood cell, or mean corpuscular hemoglobin concentration, was observed in severely cachectic Apc^Min/+^ mice when compared to C57BL/6J mice (p=0.0013) (**Figure 3F**). Additionally, increased variation in the size of red blood cells, or red cell distribution width, was observed in pre-cachectic Apc^Min/+^ and severely cachectic Apc^Min/+^ mice when compared to C57BL/6J mice (p<0.0001 and p<0.0001) (**Figure 3G**).

**Figure 4:**
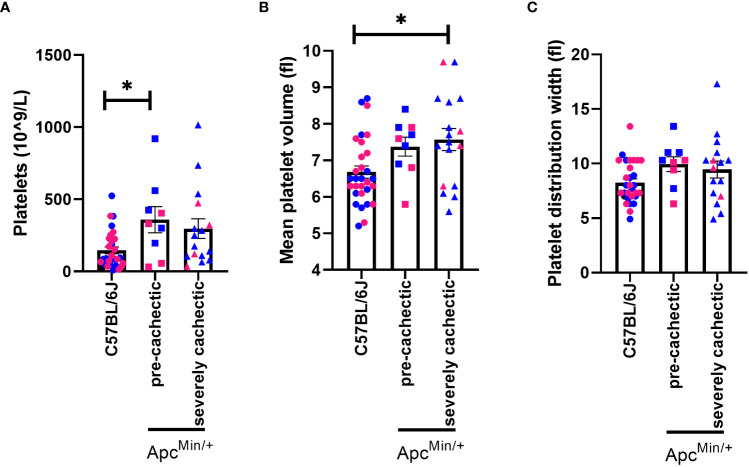
Platelet profile in male and female Apc^Min/+^ mice during cancer cachexia progression. **(A)** Platelet, **(B)** mean platelet volume and, **(C)** platelet distribution width in the blood of non-cancer control C57BL/6J (n=24), pre-cachectic Apc^Min/+^(n=9), and severely cachectic Apc^Min/+^ mice (n=17). Pink represents females, and blue represents male mice. One-way AVONA followed by a Tukey’s multiple comparisons test. *p<0.05.

Since a very high platelet count has been associated with increased risk of developing cachexia-related cancers (32) and correlated with one-year overall survival in patients with cancer cachexia (17, 18), we sought to measure platelets at different stages of cancer cachexia. We observed that platelet count in pre-cachectic Apc^Min/+^ mice was significantly elevated compared to C57BL/6J mice (**Figure 4A**, p=0.0234). There was a trend towards an increased platelet count in severely cachectic Apc^Min/+^ mice (p=0.0551). In terms of the size of platelets, severely cachectic Apc^Min/+^ mice showed an increase in mean platelet volume compared to C57BL/6J mice (**Figure 4B**, p=0.0142). Elevated mean platelet volume is indicative of platelet activation (48). There was no variation in platelet distribution observed between groups (**Figure 4C**).

### 3.3 Behavioral and physiological profiles of Apc^Min/+^ pre-cachectic male and female mice

**Figure 5:**
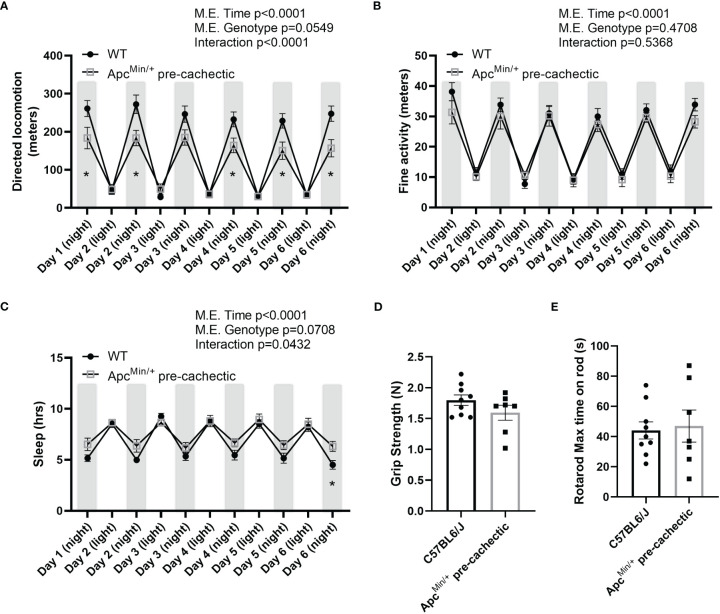
Behavioral and metabolic phenotype profile in pre-cachectic male and female Apc^Min/+^ mice over six days. **(A)** Directed locomotor activity, **(B)** stereotypic behavior (grooming, rearing, etc.), and **(C)** sleeping hours during the light and dark cycle in non-cancer control C57BL/6J (n=9) and pre-cachectic Apc^Min/+^ (n=7) mice. Mixed-effects model followed by Sidak’s multiple comparison **(D)** Grip strength and **(E)** rotarod test. Two-tailed unpaired t-test. *p<0.05.

To better understand the behavioral and physiological profile of pre-cachectic Apc^Min/+^ mice we assessed ambulatory activity, sleep patterns, forelimb strength, and neuromuscular function. Pre-cachectic Apc^Min/+^ mice showed a significant decrease in directed locomotor activity during the night cycle (active cycle) compared to C57BL/6J mice (**Figure 5A**). However, no change in stereotypical behavior (grooming, rearing, etc.) was observed between pre-cachectic Apc^Min/+^ and C57BL/6J mice (**Figure 5B**). There was a significant main effect of time for increased sleep behavior during the night cycle in pre-cachectic Apc^Min/+^ mice (**Figure 5C**). No changes were observed in grip strength and neuromuscular function between pre-cachectic Apc^Min/+^ and C57BL/6J mice (**Figures 5D, E**, p=0.1764 and p=0.8019).

Since previous studies in mice have indicated that physical inactivity and systemic metabolic dysfunction are associated with accelerated cachexia (49), we sought to investigate the impact of pre-cachexia on indirect calorimetry outcomes. We observed that pre-cachectic Apc^Min/+^ mice significantly decreased total and resting energy expenditure, oxygen consumption, and carbon dioxide production compared to C57BL/6J mice (**Table 2**). No changes were observed in mean respiratory exchange ratio, energy intake, and mean sleep. However, a significant decrease was observed in mean distance traveled in pre-cachectic Apc^Min/+^ mice compared to C57BL/6J mice.

**Table 2:** Metabolic phenotyping of pre-cachectic Apc^Min/+^ mice.

Avg. Temp	Unadjusted			ANCOVA Adjusted		
22.9 ± 0.54°C	C57BL/6J	Apc^Min/+^ pre-cachectic	p-value	C57BL/6J	Apc^Min/+^ pre-cachectic	p-value
Total EE (kcal/day)	10.02 ( ± 0.17)	8.63 ( ± 0.19)	<0.0001*	9.85 ( ± 0.17)	8.84 ( ± 0.19)	0.0017*
Resting EE (kal/hr)	0.29 ( ± 0.008)	0.25 ( ± 0.009)	0.0032*	0.29 ( ± 0.008)	0.26 ( ± 0.009)	0.0307*
O_2_ Consumption (ml/min/day)	1.42 ( ± 0.024)	1.22 ( ± 0.027)	<0.0001*	1.4 ( ± 0.024)	1.25 ( ± 0.027)	0.0018*
CO_2_ Emission (ml/min/day)	1.19 ( ± 0.025)	1.03 ( ± 0.029)	0.0014*	1.17 ( ± 0.026)	1.05 ( ± 0.029)	0.0105*
Mean Respiratory Exchange Ratio	0.83 ( ± 0.009)	0.84 ( ± 0.01)	0.6449	0.83 ( ± 0.009)	0.83 ( ± 0.011)	0.9331
Energy Intake (kcal/day)	8.8 ( ± 0.7)	8.1 ( ± 0.5)	0.4395			
Mean distance travel (meters/day)	323 ( ± 23)	246 ( ± 26)	0.0476*			
Mean sleep (hrs/day)	13.8 ( ± 0.6)	14.8 ( ± 0.4)	0.1808			

*p<0.05.

### 3.4 Skeletal muscle gene expression of immune cell markers during cancer cachexia progression in Apc^Min/+^ male and female mice

**Figure 6:**
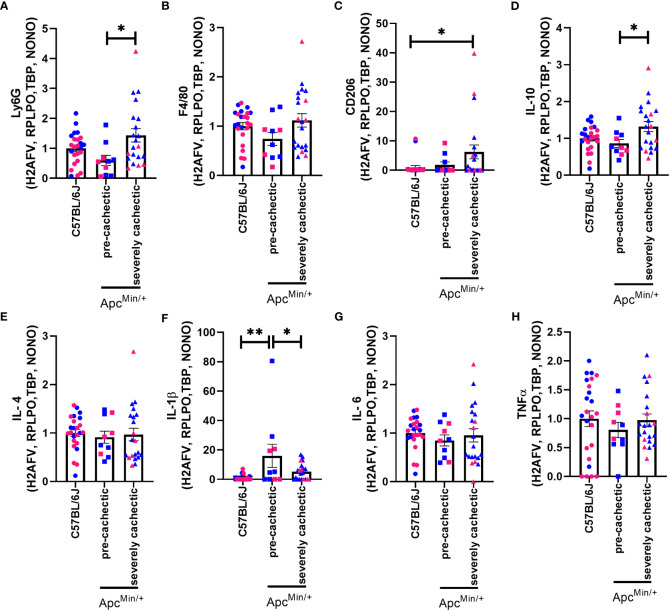
Gene expression of immune cell markers in the gastrocnemius muscle of male and female Apc^Min/+^ mice during cancer cachexia progression. **(A)** Ly6G, **(B)** F4/80, **(C)** CD206, **(D)** IL-10, **(E)** IL-4, **(F)** IL-1*β*, **(G)** IL-6, and **(H)** TNF*α* mRNA expression in non-cancer control C57BL/6J (n=24), pre-cachectic (n=10), and severely cachectic Apc^Min/+^ mice (n=21). Pink represents females, and blue represents male mice. All genes were normalized to the following reference genes H2AFV, RPLPO, TBP, and NONO. One-way AVONA followed by a Tukey’s multiple comparisons test. *p<0.05, and **p<0.01.

To determine the possible involvement of immune cells in the progression of cancer cachexia, we measured the gene expression of nonexclusive conventional immune cell markers in the skeletal muscle of non-cancer C57BL/6J mice, as well as pre-cachectic and severely cachectic Apc^Min/+^ mice. We observed a significant increase in the expression of Ly6g (neutrophil marker) in severely cachectic Apc^Min/+^ mice compared to pre-cachectic Apc^Min/+^ mice (**Figure 6A**, p=0.0178), but not C57BL/6J mice. Interestingly, severely cachectic Apc^Min/+^ mice showed an increase in CD206 (**Figure 6C**) (mannose receptor c-type 1, M2 macrophage marker, p=0.0464) and IL-10 (**Figure 6D**) (anti-inflammatory cytokine related to M2 macrophages, p=0.0216), but no change in F4/80 (a general marker of macrophages) gene expression compared to C57BL/6J mice (**Figure 6B**, p=0.1450). No difference in the gene expression of IL-4 was observed between the groups (**Figure 6E**, p=0.8874). We observed an increase in the gene expression of the pro-inflammatory cytokine IL-1β in pre-cachectic Apc^Min/+^ mice compared to C57BL/6J mice (p=0.0021) and severely cachectic Apc^Min/+^ mice (**Figure 6F**, p=0.0387). However, no changes in IL-6 and TNFα were observed between the groups (**Figures 6G, H**, p=0.6966, and p=0.6415).

### 3.5 Skeletal muscle gene expression of the TGFβ signalling pathway during cancer cachexia progression in Apc^Min/+^ male and female mice

**Figure 7:**
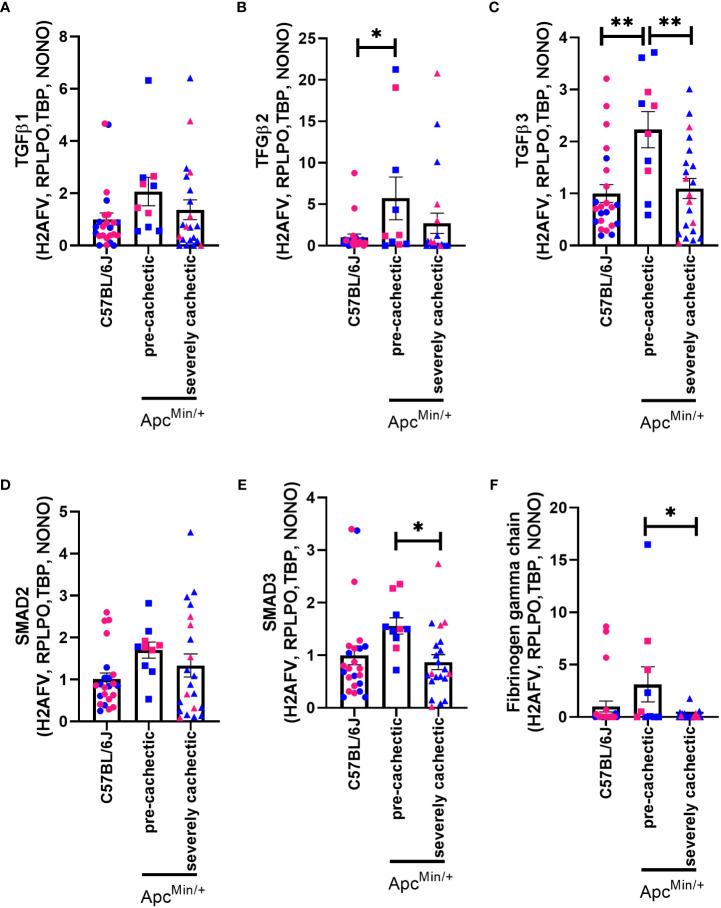
Gene expression of fibrogenic markers in the gastrocnemius muscle of male and female Apc^Min/+^ mice during cancer cachexia progression. **(A)** TGF*β*1, **(B)** TFG*β*2, **(C)** TGF*β*3, **(D)** SMAD2, **(E)** SMAD3, and **(F)** fibrinogen gamma chain mRNA expression of non-cancer control C57BL/6J (n=24), pre-cachectic (n=10), and severely cachectic Apc^Min/+^ mice (n=21). Pink represents females, and blue represents male mice. All genes were normalized to the following reference genes H2AFV, RPLPO, TBP, and NONO. One-way AVONA followed by a Tukey’s multiple comparisons test. *p<0.05, and **p<0.01.

Since platelets can promote the release of various growth factors linked to fibrosis (26), and collagen deposition has been observed in cancer cachexia (27, 50), we evaluated whether the TGFβ signalling pathway was modulated in the pre-cachectic and severely cachectic stages. No difference in the gene expression of TGFβ-1 was observed between the groups (**Figure 7A**, p=0.1816). However, a significant increase in the gene expression of TGFβ-2 and TGFβ-3 was observed in the skeletal muscle of pre-cachectic Apc^Min/+^ mice compared to C57BL/6J mice (**Figures 7B, C**, p=0.0454 and p=0.0021). Additionally, severely cachectic Apc^Min/+^ mice exhibited a significant decrease in TGFβ-3 compared to pre-cachectic Apc^Min/+^ mice (p=0.0056). We then sought to examine the downstream canonical pathways of TGFβ. No change was observed in SMAD2 between the groups (**Figure 7D**, p=0.1541). However, an increase in SMAD3 expression was observed in pre- cachectic Apc^Min/+^ mice compared to severely cachectic Apc^Min/+^ mice (**Figure 7E**, p=0.0475). Finally, we investigated the expression of fibrinogen gamma chain, a molecule essential for platelet aggregation (51), whose synthesis has been elevated in patients with cancer and cachexia (52, 53). A significant increase in the expression of fibrinogen gamma chain was observed in pre-cachectic Apc^Min/+^ mice (p=0.0312) compared to severely cachectic Apc^Min/+^ mice (**Figure 7F**).

### 3.6 Platelet profile in two models of cancer cachexia, LLC-tumor bearing mice and C26-tumor bearing mice

**Figure 8:**
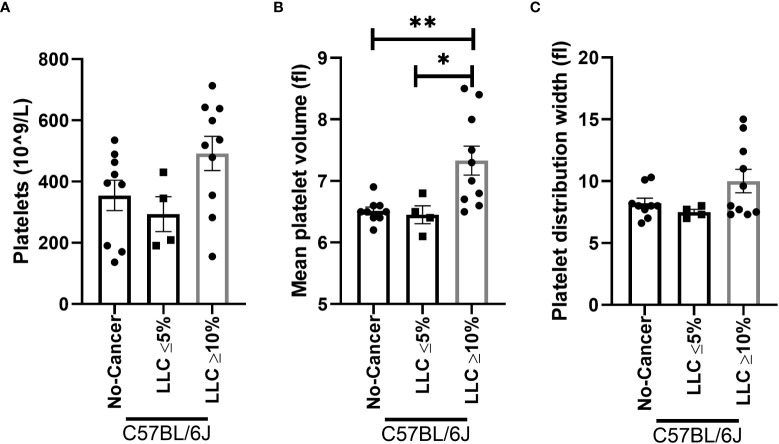
Platelet profile in male LLC-tumor bearing mice. **(A)** Platelets, **(B)** mean platelet volume, and **(C)** platelet distribution width in non-cancer control C57BL/6J mice (n=9), LLC-tumor bearing mice with less than 5% body weight loss (n=4), and LLC-tumor bearing mice with more than 10% body weight loss (n=10). One-way AVONA followed by a Tukey’s multiple comparisons test. *p<0.05, and **p<0.01.

**Figure 9:**
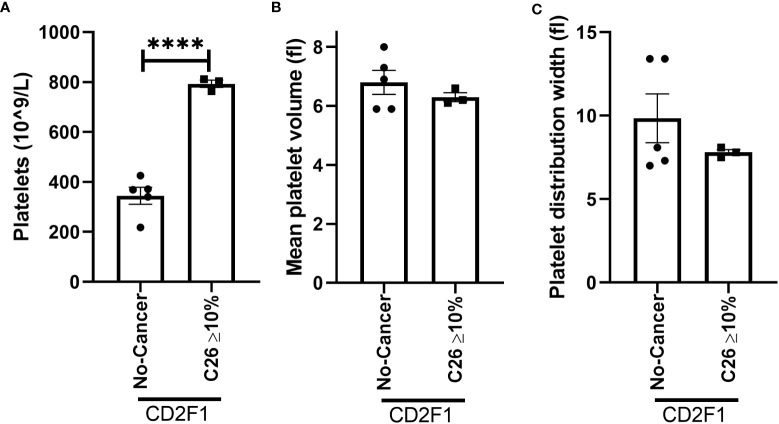
Platelet profile in male C26-tumor bearing mice. **(A)** Platelets, **(B)** mean platelet volume, and **(C)** platelet distribution width in non-cancer control CD2F1 mice (n=5) and C26-tumor bearing mice with more than 10% body weight loss (n=3). Two-tailed unpaired t-test. ****p<0.0001.

To confirm whether the increase in platelet number or platelet activation occurs in other models of muscle wasting, we examined the platelet profile in the Lewis lung carcinoma (LLC) and colon 26 adenocarcinoma (C26) models of cancer cachexia. Tumor weight in LLC-tumor bearing mice with less than five percent body weight loss (2.353 ± 0.6105 grams) and more than ten percent body weight loss (1.771 ± 0.5006 grams) were not significantly different (p=0.5129, data not shown). No difference was observed in the platelet count of LLC-tumor bearing mice (**Figure 8A**, p=0.0756). However, a significant increase in mean platelet volume was observed in severely cachectic LLC-tumor bearing mice compared to non-cancer C67BL/6J mice and LLC-tumor bearing mice with less than five percent body weight loss (**Figure 8B**, p=0.0079, p=0.0270). No significant difference was observed in platelet distribution width between the groups (**Figure 8C**, p=0.0983). In the case of C26-tumor bearing mice, an increase in platelet count was observed compared to non-cancer CD2F1 mice (**Figure 9A**, p<0.0001). There were no changes observed in mean platelet volume and platelet distribution width between C26-tumor bearing mice and non-cancer CD2F1 mice (**Figures 9B, C**, p=0.4020 and p=0.3373).

## 4 Discussion

Cachexia, a complex wasting syndrome (1, 2), significantly affects the quality of life and treatment options of cancer patients (6). It is most commonly observed in pancreatic, lung, colon, and stomach cancers (4, 33). While muscle wasting has been linked with pro-inflammatory cytokines the immunopathological aspects of the syndrome remain unexplored. Prior to cancer diagnosis, studies have noted an increase in platelet number in humans (32). Platelets can also interact with cancer cells and the tumor microenvironment, contributing to tumor progression (54–56). Additionally, cancer treatments in humans has been associated with platelet activation, indicated by high mean platelet volume (57). Elevated platelet levels are consistently correlated with a poor prognosis in cancer (28–31). Moreover, a strong association has been observed between high platelet count and decreased survival in cachectic individuals (12, 17, 18). In this study, we sought to investigate the systemic platelet profile and immunomodulatory molecules in the skeletal muscle during the progression of cancer cachexia. Our findings reveal a novel discovery that platelet elevation precedes muscle wasting and platelet activation intensifies during severely cachectic stages. Additionally, we observed that anemia and physical inactivity precede muscle mass loss. Furthermore, our study highlights the involvement of the TGFβ signaling pathway in early stages and immune cells in late stages of cancer cachexia. Overall, these findings suggest that targeting platelets could open new research avenues for cancer cachexia.

To investigate the link between platelet and muscle wasting, we analyzed blood samples using automated hematology in three established cancer cachexia models that recapitulate the human condition (58–63). Few studies have explored platelet count in rodent models with cancer cachexia (64, 65). Our study revealed increased platelet count and activation across multiple stages of cancer cachexia. Reddel *et. al*., observed elevated platelet count in C26-tumor bearing mice at late stages of cancer cachexia but not in non-cachectic mice (64). Similarly, Thomas *et. al*., reported an increase in systemic platelets (CD41^+^ cells) in LLC-tumor bearing mice and Panc02-tumor bearing mice (65). In humans, high platelets counts have been associated with renal cachexia in patients with end-stage renal disease, where the protein catabolic rate predicted platelet count (12). These findings suggest that platelets might play a role in the initiation and progression of muscle wasting.

Besides hemostasis, platelets play a critical role in inflammation (37). They interact with immune cells such as macrophages and neutrophils (24, 25). In severely cachectic mice, we observed an increase in neutrophils and a decrease in lymphocytes, with no changes in monocytes. These changes in white blood cells have been previously reported in cancer patients (66–70). Similarly, we found an increase in the expression of Ly6G, CD206, and IL-10 in the skeletal muscle of severely cachectic mice. Macrophage recruitment has been observed in the skeletal muscle of severely cachectic patients (27). Depletion of neutrophils in rodents slows down muscle wasting in mice with pancreatic and lung cancer (25, 71). We also observed an increase in IL-1β in the skeletal muscle of pre-cachectic mice, but not at the severely cachectic stage. IL-1β can be secreted from various cells including monocytes, macrophages, neutrophils, and platelets (72–75). IL-1β has been implicated in cancer cachexia in humans (76). It is possible that the increase in platelets leads to an increase in IL-1β. Conducting future experiments to deplete and adoptively transfer platelets could provide valuable insights into the role of platelets in modulating neutrophils, macrophages, and lymphocytes in cancer cachexia.

Other evidence that sets platelets as an important contributor to cancer cachexia is their capacity to promote latent TGFβ activation (77). In both pre-cachexia and cachexia stages, elevated expression of the canonical signaling pathway of TGFβ1 has been observed in the plasma of cancer patients (67). Similarly, the skeletal muscle of pancreatic cachectic patients exhibit elevated transcripts involved in TGFβ activation and the develop of fibrosis, which correlates with decreased survival (27). In our study, we observed an increase in TGFβ2, TGFβ3, and SMAD3, but not TGFβ1, in the skeletal muscle of pre-cachectic mice. However, an increase in TGFβ1 has been observed in early cachexia in LLC-tumor bearing mice (50). Overexpression and administration of TGFβ2 has been shown to induce cachexia in mice, rats, and humans (78–80). While extensive research has been conducted on the role of TGFβ1 in other wasting conditions such as muscle dystrophy (50, 81–89), the contribution of TGFβ2 and TGFβ3 to cancer cachexia remains unexplored.

We found anemia in Apc^Min/+^ mice starting at the pre-cachectic stage and continuing into the severely cachectic stage. Baltgalvis et al., demonstrated that anemia occurs before reductions in wheel running performance in Apc^Min/+^ mice (90). Anemia has also been observed in patients with cancer cachexia and has been shown to predict physical activity (91). In our study, we observed a decrease in direct locomotor activity in Apc^Min/+^ mice, even without changes in grip strength and rotarod performance. Anemia and physical inactivity have been observed in other cancer cachexia models, such as LLC- and C26-tumor bearing mice (49, 92, 93). Therefore, we speculate that physical inactivity may be caused by anemia. However, others have attributed the decline in activity performance to cancer-related fatigue (92, 94). Whether anemia in cancer cachexia reduces oxygen levels in skeletal muscle or the brain, leading to physical inactivity and fatigue, remains unknown.

A limitation of this study is the lack of power to test for sex differences. In this study, no assessments were performed to determine the estrus cycle in female mice nor were hormonal concentrations measured in either sex. Previous observations have indicated a decline in ovarian function in Apc^Min/+^ female mice (95), while pre-clinical and clinical studies have suggested potential sex differences during the initiation and progression of cancer cachexia (42, 50, 96–99). Some studies have separated males and females to test the data, but this approach does not determine whether the sexes responded differently. Others have tested sex as a factor in a factorial design without including if the interaction was significant. Additionally, studies have not accounted for the differences in hormonal status between females and also males. Remarkably, approximately seventy percent of biological science manuscripts have reported sex differences (sexual dimorphism, sex-dependent, and sex-specific) without statistically comparing the sexes (100). Consequently, further studies are necessary to investigate whether sex differences exist in cancer cachexia and whether hormonal status contributes to these differences.

While our understanding of the pro-inflammatory cytokines, IL-6 and TNF-α leading to cancer cachexia has been established, the immunopathogenesis of muscle wasting has been overlooked. In general, we sought to investigate the blood cellular elements and immune adaptations throughout the progression of cancer cachexia, shedding new light on the role of early platelet presence and immune cell infiltration in this syndrome. Our findings reveal that platelet count is elevated prior to cachexia development in Apc^Min/+^ mice and can become activated during its progression. Furthermore, we observed an increased expression of TGFβ2, TGFβ3, and SMAD3 in the skeletal muscle of pre-cachectic Apc^Min/+^ mice. In severely cachectic mice we noted elevated gene expression related to neutrophils and anti-inflammatory macrophages. Additionally, we found an increase in anemia associated with physical inactivity. Our behavioral and metabolic phenotyping findings suggest that pre-cachectic Apc^Min/+^ mice decrease physical activity to conserve energy. Overall, our findings demonstrate altered platelet status during early and late stages of cachexia, paving the way for further investigation of platelets in the field of cancer cachexia.

## Data availability statement

The original contributions presented in the study are included in the article/supplementary materials, further inquiries can be directed to the corresponding author/s.

## Ethics statement

The animal study was approved by Institutional Animal Care and Use Committee (IACUC) of the University of South Carolina. The study was conducted in accordance with the local legislation and institutional requirements.

## Author contributions

KV: Conceptualization, Data curation, Formal Analysis, Funding acquisition, Project administration, Resources, Supervision, Writing – review & editing. PC: Conceptualization, Data curation, Formal Analysis, Writing – original draft, Writing – review & editing. CU: Conceptualization, Data curation, Formal Analysis, Writing – review & editing. EP: Data curation, Formal Analysis, Writing – review & editing. AA: Writing – original draft, Writing – review & editing. AB: Writing – original draft, Writing – review & editing. EJ: Data curation, Formal Analysis, Writing – review & editing. AA: Data curation, Formal Analysis, Writing – review & editing. MH: Data curation, Formal Analysis, Writing – review & editing. BV: Data curation, Formal Analysis, Writing – review & editing. TC: Data curation, Formal Analysis, Writing – review & editing. EM: Data curation, Formal Analysis, Funding acquisition, Resources, Supervision, Writing – review & editing. RE: Conceptualization, Data curation, Funding acquisition, Resources, Supervision, Writing – review & editing.
